# Supplementary material for: Causal Relationship between Aging and Anorexia Nervosa: A White-Matter-Microstructure-Mediated Mendelian Randomization Analysis
Source: Biomedicines. 2024 Aug 16;12(8):1874. doi: 10.3390/biomedicines12081874 (PMC11351342; doi:10.3390/biomedicines12081874)
Supplement: Supplementary file 1 [file biomedicines-12-01874-s001.zip › Supplementary Figure/Figure S4/Singe SNP plot of MR analysis for TL to ubm-b-1608.pdf]

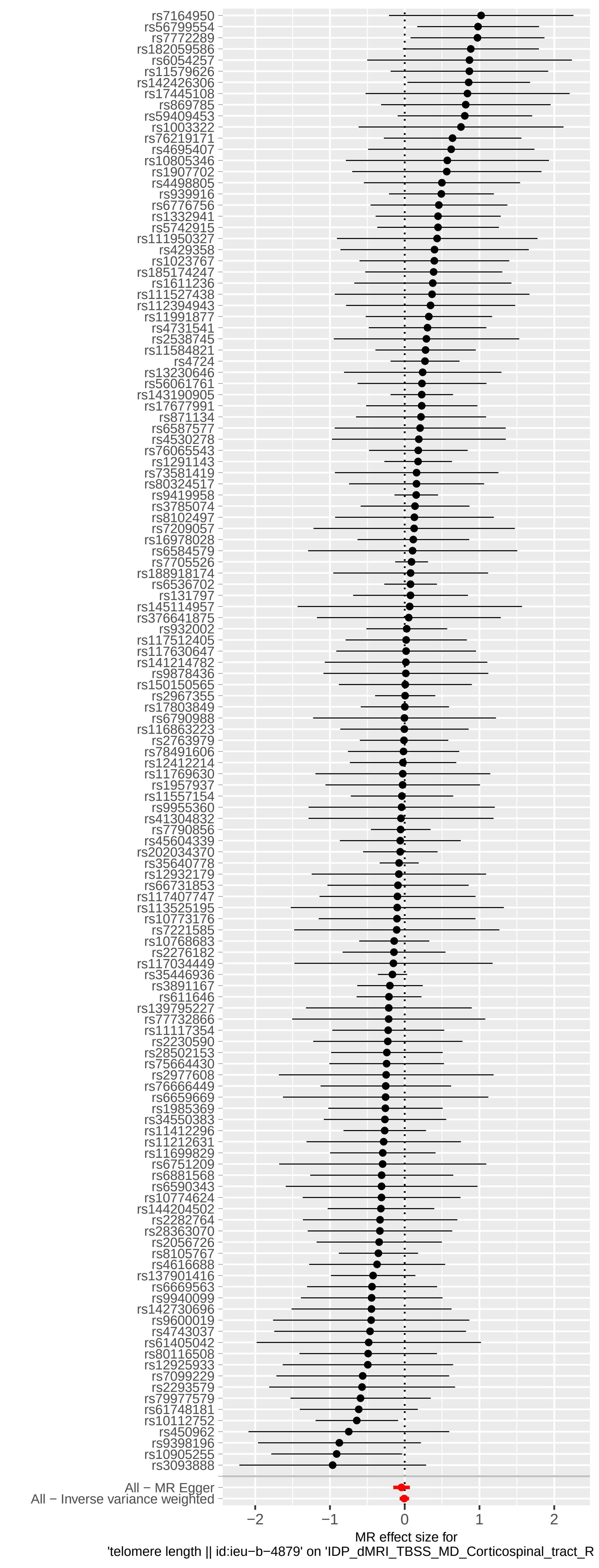
rs7164950
rs56799554
rs7772289
rs182059586
rs6054257
rs11579626
rs142426306
rs17445108
rs869785
rs59409453
rs1003322
rs76219171
rs4695407
rs10805346
rs1907702
rs4498805
rs939916
rs6776756
rs1332941
rs5742915
rs111950327
rs429358
rs1023767
rs185174247
rs1611236
rs111527438
rs112394943
rs11991877
rs4731541
rs2538745
rs11584821
rs4724
rs13230646
rs56061761
rs143190905
rs17677991
rs871134
rs6587577
rs4530278
rs76065543
rs1291143
rs73581419
rs80324517
rs9419958
rs3785074
rs8102497
rs7209057
rs16978028
rs6584579
rs7705526
rs188918174
rs6536702
rs131797
rs145114957
rs376641875
rs932002
rs117512405
rs117630647
rs141214782
rs9878436
rs150150565
rs2967355
rs17803849
rs6790988
rs116863223
rs2763979
rs78491606
rs12412214
rs11769630
rs1957937
rs11557154
rs9955360
rs41304832
rs7790856
rs45604339
rs202034370
rs35640778
rs12932179
rs66731853
rs117407747
rs113525195
rs10773176
rs7221585
rs10768683
rs2276182
rs117034449
rs35446936
rs3891167
rs611646
rs139795227
rs77732866
rs11117354
rs2230590
rs28502153
rs75664430
rs2977608
rs76666449
rs6659669
rs1985369
rs34550383
rs11412296
rs11212631
rs11699829
rs6751209
rs6881568
rs6590343
rs10774624
rs144204502
rs2282764
rs28363070
rs2056726
rs8105767
rs4616688
rs137901416
rs6669563
rs9940099
rs142730696
rs9600019
rs4743037
rs61405042
rs80116508
rs12925933
rs7099229
rs2293579
rs79977579
rs61748181
rs10112752
rs450962
rs9398196
rs10905255
rs3093888
All − MR Egger
All − Inverse variance weighted
−2
−1
0
1
2
MR effect size for
'telomere length || id:ieu−b−4879' on 'IDP_dMRI_TBSS_MD_Corticospinal_tract_R
